# Development of cancer-associated fibroblasts-targeting polymeric nanoparticles loaded with 8-*O*-methylfusarubin for breast cancer treatment

**DOI:** 10.1016/j.ijpx.2024.100294

**Published:** 2024-10-17

**Authors:** Kamonlatth Rodponthukwaji, Suyanee Thongchot, Suttikiat Deureh, Tanva Thongkleang, Mattika Thaweesuvannasak, Kornrawee Srichan, Chatchawan Srisawat, Peti Thuwajit, Kytai T. Nguyen, Kwanruthai Tadpetch, Chanitra Thuwajit, Primana Punnakitikashem

**Affiliations:** a Department of Biochemistry, Faculty of Medicine Siriraj Hospital, Mahidol University, Bangkok 10700, Thailand; b Siriraj Center of Research Excellence in Theranostic Nanomedicine, Research Department, Faculty of Medicine Siriraj Hospital, Mahidol University, Bangkok 10700, Thailand; c Department of Immunology, Faculty of Medicine Siriraj Hospital, Mahidol University, Bangkok 10700, Thailand; d Siriraj Center of Research Excellence for Cancer Immunotherapy (SiCORE-CIT), Research Department, Faculty of Medicine Siriraj Hospital, Mahidol University, Bangkok 10700, Thailand; e Department of Bioengineering, University of Texas at Arlington, Arlington, TX 76019, USA; f Division of Physical Science and Center of Excellence for Innovation in Chemistry, Faculty of Science, Prince of Songkla University, Hat Yai, Songkhla 90110, Thailand

**Keywords:** Cancer-associated fibroblasts, 8-*O*-methylfusarubin, PLGA, Drug delivery system, Tumor microenvironment

## Abstract

Cancer-associated fibroblasts (CAFs) are abundant stromal cells residing in a tumor microenvironment (TME) which are associated with the progression of tumor. Herein, we developed novel CAFs-targeting polymeric nanoparticles encapsulating a synthetic 8-*O*-methylfusarubin (OMF) compound (OMF@NPs-anti-FAP). Anti-FAP/fibroblast activation protein antibody was employed as a CAFs-targeting ligand. The physicochemical properties of the synthesized nanomaterials were firstly investigated with various techniques. The cytocompatibility of polymeric nanoparticles (NPs) was elicited through cell viability of CAFs and human breast epithelial cells, MCF-10A. Additionally, the anti-FAP-conjugated NPs displayed different degrees of cellular internalization regarding the FAP expression level on the CAFs' surface. However, CAFs exposed to NPs containing OMF demonstrated significant cell death which were associated with the apoptotic pathway as confirmed by caspase-3/7 activity. Upon OMF@NPs-anti-FAP treatment, an enhanced toxicity was clearly observed in 3D spheroid models. High FAP-expressed PC-B-132CAFs demonstrated a high percentage of cell death compared to other cells with a low level of FAP expression analyzed by flow cytometry (e.g. MCF-10A, HDFa, and PC-B-142CAFs). This result emphasized the importance of anti-FAP antibody as a targeting ligand. These findings suggest that the fabricated nanosystem of OMF-loaded polymeric NPs with CAFs' high specificity holds a potential NP-based platform for improvement in breast cancer treatment.

## Graphical abstract

**Figure:**
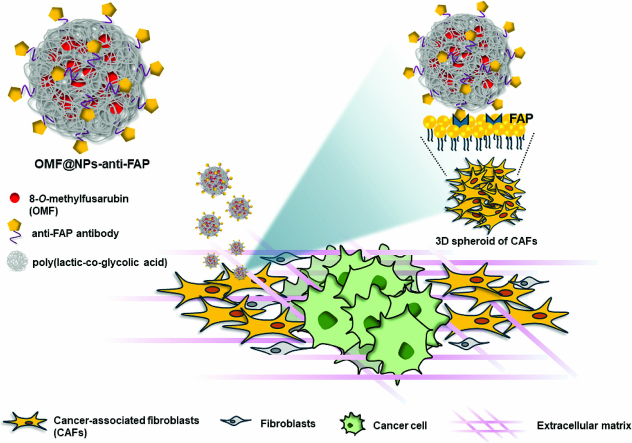

## 1 Introduction

Annually, 12.5 % of all newly diagnosed cancers were globally attributed to breast cancer. Also, it makes breast cancer the most common cancer among women in the U.S., accounting for 30 % of all new cases(Breastcancer.org, 2023). Several approaches including surgery, chemotherapy, radiation therapy, etc. are optionally adopted to treat the cancer (Service, N.H, 2022). Despite effective chemotherapy, toxicity caused by off-target and high-dose usage of anticancer drugs remains a burden for cancer treatment (Rao et al., 2019; Tacar et al., 2013). Thus, enormous efforts have been made to improve strategies for cancer treatment.

The tumor microenvironment (TME) of breast cancer is constructed of several components including tumor cells, immune cells, endothelial cells, and cancer-associated fibroblasts (CAFs) (Li et al., 2021). Among those, CAFs have been considered as one of the most significant mediators that cause cancer cell invasion (Glentis et al., 2017), radioresistance (Guo et al., 2023), and an immunosuppressive microenvironment (Monteran and Erez, 2019). Communications between CAFs and cancer cells through secreted cytokines and chemokines can also support a pro-tumorigenic environment (Yoshida, 2020). Our previous work demonstrated that IL-6 secreted from CAFs could induce DOX-resistance cells (Chuangchot et al., 2023). Therefore, inhibition of CAFs' progression could be an alternative approach for breast cancer treatment. Traditionally, fibroblast activation protein (FAP) is one of the most common biomarkers which is highly expressed on the activated CAFs' surface (Xin et al., 2021). Thus, a constructively therapeutic approach, thereby targeting FAP-positive CAFs would be an effective strategy for curing breast cancer.

As CAFs are our prime target, suppressing proliferation of CAFs with cytotoxic agents can provide effective outcomes. Although several chemically synthetic compounds, such as alkylating agents, have shown potentially preventive effects, side effects caused by its high toxicity play undesirable outcomes. Alternatively, natural substances offer interesting benefits over traditional chemotherapy with less toxicity. The pyranonaphthoquinones are metabolites that can be isolated from bacteria, fungi, and microorganisms. With the main skeleton of naphtho[2,3-*c*]pyran-5,10-dione, the pyranonaphthoquinones and their derivatives could express diverse bioactivities, including antibacterial, antimalarial, and anticancer activities (Dang Thi et al., 2015; Lemos et al., 2018; Saisin et al., 2023). Previously, a bioactive compound, 8-*O*-methylfusarubin (OMF), which was isolated from a seagrass-derived fungus *Pestalotiopsis* sp. PSU-ES180 and its first synthesis reported by our group, showed great potential in inhibiting MCF-7 proliferation (Vijitphan et al., 2019). Despite its efficacy in prohibiting cell proliferation, aqueous insolubility of the therapeutic agent may hamper its clinical application as an insoluble drug could perform poor permeability, and ease of elimination from the body system. Accordingly, several administrations or high-dose usages are required in order to reach an effective dose, leading to suboptimal drug delivery (Mady et al., 2023).

To enhance the bioavailability of the insoluble drug, a nanoparticle (NP)-based drug delivery system has been extensively employed. Several types of nanoparticles have been utilized in drug delivery platforms, such as mesoporous silica nanoparticles (Hazeri et al., 2022), liposomes (Dana et al., 2023; Tang et al., 2023), and polymeric nanoparticles (Misiak et al., 2023). Among those, polymeric nanoparticles, particularly poly(lactic-*co*-glycolic acid) or PLGA, are of interest due to their biocompatibility, biodegradability, and ease of surface functionalization (Calzoni et al., 2019; Elmowafy et al., 2019; Shipunova et al., 2021). Recently, we developed a therapeutic polymeric-based platform by encapsulating therapeutic small interfering RNA (siRNA). The therapeutic effect of loaded siRNA was elicited, demonstrating the reduction of cancer cells' viability (Punuch et al., 2022; Rodponthukwaji et al., 2024). Additionally, TKD peptide-conjugated polymeric nanoparticles could deliver antitumor peptides specifically to breast tumor cells (Mohan and M.M., Kumar TRS, Kumar GSV, 2022). Due to their ability to carry therapeutic drugs and target ligands, the utilization of polymeric nanoparticles as drug carriers could be beneficial in this work.

Therefore, to overcome the drawbacks of insoluble OMF drugs, we, herein, firstly developed OMF-loaded polymeric nanoparticles conjugated with anti-FAP monoclonal antibody as a new drug delivery platform with capacity for CAFs' targeting. The physiochemical and biological activities of the synthesized nanoplatforms were comprehensively studied. A significant role of synthetic compound OMF against CAFs was investigated through the cell viability and caspase-3/7 activity. To verify the targeting capability of the antibody-conjugated nanosystem, cellular internalization was conducted and analyzed by flow cytometry. Also, the benefit of targeting the moiety of the anti-FAP antibody was clearly observed via an enhanced cytotoxic effect in 3D spheroid models regarding the different levels of FAP-expressed cells.

## 2 Materials and Methods

### 2.1 Chemicals and biological reagents

All chemicals and reagents were used as received without further purification. 8-*O*-Methylfusarubin was synthesized from 5-bromovanillin using our reported protocol (Vijitphan et al., 2019). Resomer® RG 503H, Poly(D,l-lactide-*co*-glycolide or PLGA) with MW of 24,000–38,000 g/mol, poly(vinyl alcohol) with average MW of 31,000–50,000 (PVA), dichloromethane (DCM, ≥ 99.8 %), dimethylsulfoxide (DMSO), N-(3-Dimethylaminopropyl)-N′-ethylcarbodiimide hydrochloride (EDC), N-Hydroxysulfosuccinimide sodium salt (sulfo-NHS, ≥ 98 %), phosphate-buffered saline (PBS), and 2-(N-Morpholino)ethanesulfonic acid sodium salt (MES, ≥ 99 %) were purchased from Sigma-Aldrich (St. Louis, MO, USA). CellTiter-Blue® Cell Viability Assay, Apo-ONE® Homogeneous Caspase-3/7 Assay were acquired from Promega (Madison, WI, USA). Dulbecco's Modified Eagle Medium Nutrient Mixture F-12 (DMEM/F-12), Fetal bovine serum (FBS), and penicillin–streptomycin (Pen-Strep) were received from Gibco (Grand Island, NY, USA). CellTracker™ Green CMFDA (5-chloromethylfluorescein diacetate) Dye was a product of Thermo Fisher Scientific (Waltham, MA, USA). Corning matrigel matrix was bought from Corning (NY, USA). Bio-Rad protein assay dye was purchased from Bio-Rad (Hercules, CA, USA).

### 2.2 Synthesis of unloaded and drug loaded-polymeric nanoparticles

Unloaded polymeric nanoparticles (NPs) were synthesized using a standard emulsion method described previously with some modifications (Punuch et al., 2022). Firstly, 20 mg of PLGA was dissolved in DCM. Next, the PLGA solution was added dropwise into a solution containing 2 % *w*/*v* PVA. Then, the mixture was sonicated by using an ultrasonic probe sonicator (Sonics and Materials Inc., Newtown, CT, USA) with 30 % amplitude with the pulse cycling on and off every 1 s for 10 min on an ice bath. After that, the nanoparticle suspension was subsequently stirred at room temperature to remove the organic solvent. Finally, the particles, NPs, were obtained using Sorvall RC6+ centrifuge (Thermo Fisher Scientific, Asheville, NC, USA) at 12,000 rpm at 10 min and resuspended in water. Lyophilized particles could also be carried out.

For the preparation of 8-*O*-methylfusarubin-loaded polymeric nanoparticles or OMF-NPs, a similar protocol was carried out. Firstly, a solution of OMF in DMSO was added dropwise into a solution of PLGA. Then, the first emulsion was obtained by sonicating the mixture with an ultrasonic probe sonicator. Then, the first emulsion was added dropwise into a solution containing 2 % w/v PVA, and the same protocol described above was applied to produce the second emulsion. The pellet of nanoparticle was collected and supernatant containing unencapsulated OMF was collected by centrifugation at 12,000 rpm at 10 min. Then, the final product of OMF@NPs powder was then obtained by lyophilization.

### 2.3 Antibody functionalization of polymeric nanoparticles

The lyophilized NPs were further conjugated with human fibroblast activation protein alpha/FAP antibody (MAB3715, R&D Systems, Minneapolis, MN, USA). Briefly, polymeric nanoparticles were firstly activated with carbodiimide and sulfo-NHS coupling reagents in pH 4.7 of 0.45 mM MES buffer for 2 h. Then, the activated NPs were recovered by centrifugation. The activated NPs were then conjugated with anti-FAP in sterile PBS pH 7.4 containing antibody with the final concentration of 8 μg/mL. The mixture was carried out at 4 °C overnight. The final product was obtained by centrifugation. % conjugation of antibody was quantified by using Bio-Rad protein assay dye (Hercules, CA, USA).

### 2.4 Characterization of the synthesized nanoparticles

The hydrodynamic size and zeta potential of the synthesized nanoparticles were measured by a Zetasizer (Malvern Instruments, Worcestershire, UK). The morphology determination was investigated by field emission scanning electron microscopy (FESEM) (SU8030, Hitachi, Ltd., Japan) and transmission electron microscopy (TEM) (JEM2100 plus, JEOL, Japan). A UV–Vis spectrophotometer (Bio-Tek Instrument Inc., Winooski, VT, USA) was used to confirm the successful loading of the OMF drug. The surface functionalization of nanoparticles was also determined by using Fourier-transform infrared spectroscopy (FTIR). The percentage of encapsulation efficiency of payload (%EE) and the percentage of drug loading (%DL) was obtained by a direct method by dissolving the drug-loaded NPs in DMSO. The amount of loaded drug was correlated to the absorbance measured at 490 nm by using Multi-Detection Microplate Readers (Bio-Tek Instrument Inc., Winooski, VT, USA). The drug release profile of OMF from OMF@NPs-anti-FAP was performed by using the centrifugation method. The released media was replaced with fresh media of the same volume and the amount of loaded OMF was determined by measuring the absorbance at 490 nm. The percentage of conjugated antibody was quantified by using Bio-Rad protein assay which was based on the Bradford protein assay.

### 2.5 Cell line and cell culture

The in-house primary culture breast CAFs, PC-B-132CAFs and PC-B-142CAFs, MCF-10A, a normal mammary epithelial cell (ATCC, USA), was cultured in Dulbecco's Modified Eagle Medium Nutrient Mixture F-12 (DMEM/F-12) supplemented with 10 % fetal bovine serum (FBS) (Invitrogen, USA) and 100 IU/mL penicillin-streptomycin at 37 °C in an incubator with 5 % CO_2_. MCF-10A was cultured in DMEM supplemented with 5 % horse serum (Invitrogen Corporation), 100 U/mL of penicillin and 100 μg/mL of streptomycin, 20 ng/mL EGF (PeproTech), 0.5 mg/mL hydrocortisone (Sigma-Aldrich), 100 ng/mL cholera toxin (Sigma-Aldrich), and 10 μg/mL insulin (Sigma-Aldrich). Primary Human Dermal Fibroblasts adult (HDFa, ATCC, USA) were cultured in DMEM supplemented with 10 % fetal bovine serum (FBS) (Invitrogen, USA) and 100 IU/mL penicillin-streptomycin. All the cells were maintained in a 5 % CO_2_ incubator at 37 °C.

### 2.6 Cytotoxicity study

The cytotoxicity of unloaded nanoparticles (NPs), free monoclonal antibody (anti-FAP), anti-FAP conjugated NPs (NPs-anti-FAP), OMF@NPs, and OMF@NPs-anti-FAP were tested on different types of cell lines, PC-B-132CAFs, PC-B-142CAFs, and MCF-10A. The cells (1 × 10^4^ cells/well) were seeded in a 96-well plate and left to adhere overnight. Next, the samples with different concentrations were added into the wells. After 24 h of incubation, the viability of the cells was quantified by using CellTiter-Blue® Cell Viability Assay following the manufacturer's protocol. The fluorescent signal was measured at Ex485/Em530 nm by multi-detection microplate readers (Bio-Tek Instrument Inc., Winooski, VT, USA).

### 2.7 Cellular internalization

PC-B-132CAFs and PC-B-142CAFs (1 × 10^4^ cells/well) were seeded in a 48-well plate and allowed to adhere overnight. Then, the samples, including Cou6@NPs, Cou6@NPs-anti-FAP were tested on those cells at 30 min and 1 h. Herein, coumarin-6 (Cou6) was used as a representative of OMF. The treated cells were washed with PBS and detached with Trypsin-EDTA (0.25 %) (Gibco, USA) followed by the addition of trypsin neutralizing solution. Centrifugation was performed at 1500 rpm for 3 min, and the supernatant was discarded. The pellet was resuspended in FACs buffer. The cellular internalization was determined on a flow cytometer (BD FACSCelesta™, BD Bioscience, San Jose, CA, USA). Flow cytometric data were analyzed using FlowJo V10 software (BD Bioscience, San Jose, CA, USA).

### 2.8 Caspase-3/7 activity

For the caspase-3/7 activity assay, a consecutive protocol was performed after the cell viability assay. The mixture of caspase 3/7 substrate and buffer was prepared according to the manufacturer's protocol of the Apo-ONE® Homogeneous caspase-3/7 assay (Promega, Madison, WI, USA), and was then added into each well with the volume ratio of 1:1 of CellTiter-Blue and caspase 3/7 mixture. The mixture was then incubated in the dark for 30 min at 37 °C. The fluorescence intensity was measured at Ex485/Em530 nm by multi-detection microplate readers (Bio-Tek Instrument Inc., Winooski, VT, USA).

### 2.9 Determination of FAP expression by flow cytometry

The intrinsic expression of surface FAP was assessed. MCF-10A, HDFa, PC-B-132CAFs, and PC-B-142CAFs were stained with 1:100 rabbit anti-FAP (ab53066, Abcam) for 1 h at room temperature. The cells were washed twice with PBS and incubated 1:200 donkey anti-rabbit IgG, Alexa Fluor 488 (A21206, Life Technology) for 1 h at room temperature. The stained cells were washed twice with PBS. The cells were examined by CytoFLEX (Beckman Coulter). The data were analyzed using FlowJo 10 software (FlowJo, LLC, Ashland, OR).

### 2.10 Cytotoxicity (% killing) assays

Cytotoxicity assay was conducted by using a three-dimensional (3D) spheroid model. In brief, a total number of 1 × 10^3^ cancer cells were first stained with CellTracker™ Green CMFDA (5-chloromethylfluorescein diacetate) Dye (Thermo Fisher Scientific, Waltham, MA) and then seeded into an ultra-low attachment 96-well round-bottomed plate (Corning, NY, USA) containing 2.5 % Corning matrigel matrix (Corning, NY, USA). To generate a single spheroid, the plate was centrifuged at 300 ×*g* at 4 °C for 3 mins and then cultured for 3 days. Treatment medium including NPs, OMF, OMF@NPs, and OMF@NPs-anti-FAP containing 1 μg/mL propidium iodide (PI) were added to the spheroid. After co-culturing for 24 h, live cancer cells, which were stained with CMFDA; and dead cancer cells stained with PI were captured by a fluorescence inverted microscopy and CellSense Standard program version 1.15 (Olympus). ImageJ (NIH) software was used to quantify the change in mean fluorescence intensity (MFI). Cytotoxicity (% killing) was calculated by the following formula:

$$\mathrm{Cytotoxicity}\;\left(\%\right)=\left[\frac{\mathrm{experimental}\;\mathrm{MFI}-\mathrm{spontaneous}\;\mathrm{MFI}}{\mathrm{spontaneous}\;\mathrm{MFI}}\right]\times 100$$

Experimental MFIs were MFI of sample spheroids when co-cultured with treatment.

Spontaneous MFIs were MFI of sample spheroids when co-cultured without treatment.

### 2.11 Statistical analysis

All results were presented as mean ± SD (*n* = 3). The statistical analysis of multiple group comparisons was tested by one-way ANOVA followed by Tukey's post hoc test using the GraphPad Prism version 8 (GraphPad Software).

## 3 Results and Discussion

### 3.1 Characterization of nanoparticles

**Fig. 1:**
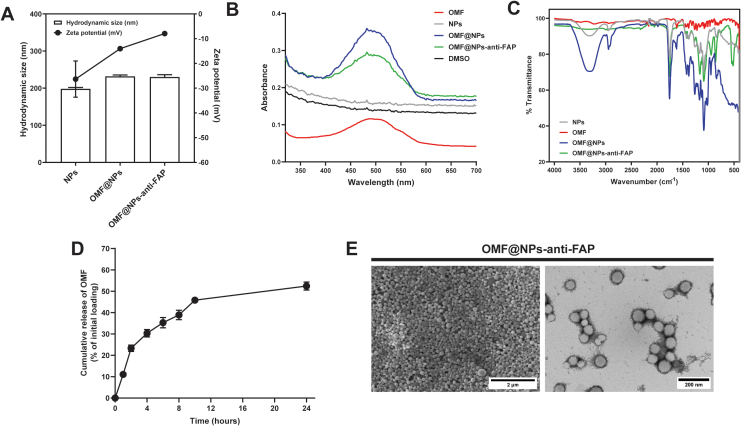
Physicochemical properties of the synthesized polymeric NPs were analyzed using different techniques. (A) Hydrodynamic size and zeta potential of the synthesized NPs, OMF@NPs, and OMF@NPs-anti-FAP determined by Zetasizer. (B) The OMF loading capability of the polymeric NPs were determined by UV–Vis spectrophotometer. (C) Conjugation of the anti-FAP monoclonal antibody onto NPs' surface determined by FTIR technique. (D) The OMF release profile of OMF@NPs-anti-FAP in completed media at 37 °C. (E) Morphology analysis of OMF@NPs-anti-FAP using FESEM and TEM techniques, respectively.

In this work, we successfully produced polymeric NPs. The synthesized nanoparticles including unloaded NPs, OMF-loaded NPs (OMF@NPs), and anti-FAP-conjugated OMF@NPs (OMF@NPs-anti-FAP) demonstrated hydrodynamic sizes of 198.17± 3.75 nm, 231.93 ± 3.65 nm, and 230.10 ± 6.21 nm, respectively (Fig. 1A). The similarity in size between the unloaded and loaded NPs suggested that OMF did not alter the physical properties of the core material. Additionally, the size of the conjugated nanoparticles was doubly increased, implying the successful conjugation of anti-FAP monoclonal antibody onto the NPs' surface. Zetasizer also revealed a change in the zeta potential of the materials after surface modification. Unloaded NPs showed a strongly negative charge of −26.33 ± 7.32 mV. However, the incorporation of OMF could slightly reduce its negativity (−14.03 ± 0.80 mV). On the contrary, the negatively charged OMF@NPs were partially covered by the presence of the anti-FAP antibody, providing the increased positivity of −7.95 ± 0.42 mV in OMF@NPs-anti-FAP. A similar result was observed in previous work (Gandhi and Shende, 2024; Lee et al., 2021). Nevertheless, the hydrodynamic size of OMF@NPs and OMF@NPs-anti-FAP showed no significant difference, while the zeta potential exhibited a notable shift. This phenomenon can be attributed to the low amount of antibody conjugation, which was only 0.14 mass % of the total nanoparticles (Saha et al., 2024). The change in zeta potential indicates that even a small modification with the anti-FAP antibody can impact the surface charge characteristics of the nanoparticles, potentially influencing their interaction with target cells. Therefore, the reduced negative charge after antibody conjugation could confirm the successful conjugation of the targeting ligand, anti-FAP antibody, onto our nanoparticles' surface.

Furthermore, the presence of OMF in NPs was also investigated by using a UV–Vis spectrophotometer. The UV–Vis spectra shown in Fig. 1B indicated that the OMF compound was successfully encapsulated inside the NPs with a high percentage of encapsulation efficiency (EE) of 81.63 ± 2.58 %. The percentage of drug loading (%DL) of 15.23 ± 0.48 % was attained. Both %EE and %DL reported in this work are comparable to the previous reports (Sun et al., 2015; Zhang et al., 2018). The OMF@NPs and OMF@NPs-anti-FAP showed similar patterns as the free OMF, while unloaded NPs and DMSO, a solvent used to dissolve OMF, remained plateau. This result suggested that polymeric nanoparticles were capable of carrying the payload.

In addition to size and zeta potential determination, the Fourier-transform infrared spectroscopy (FTIR) technique was exploited to investigate the antibody modification of the NPs. As shown in Fig. 1C, a strong signal of the peak at about 3000 cm^−1^ of OMF@NPs-anti-FAP, which represented C—H stretching vibrations of CH-, CH_2_-, and CH_3_- groups, decreased. However, herein, significant peaks that were related to amide I and amide II could not be identified as those were obscured by intense signals of OMF. However, a sharp N—H deformation peak around 600 cm^−1^ was clearly observed, implying the successful conjugation of the anti-FAP monoclonal antibody (Lou et al., 2012). The percentage of antibody conjugation was quantified as 43.78± 14.18 % determined by Bradford protein assay.

To utilize nanoparticles as drug carriers, the capability of the nanomaterial in releasing the therapeutic OMF drug was also performed in completed media at 37 °C (Fig. 1D). The established OMF@NPs-anti-FAP demonstrated a burst release characteristic at the first 10 h by releasing about 45.84± 1.24 % of OMF followed by the sustained-release character over 24 h, releasing about 52.44 ± 1.88 % of OMF. A high percentage of the drug was released in a burst-release manner within the first 10 h. The burst-release phenomenon is reportedly proportional to the high encapsulated OMF (%DL of 15.23 ± 0.48 %). The high amount of OMF could be accumulated at the near surface of the polymeric matrix, facilitating the release of the encapsulated drug (Gutiérrez-Valenzuela et al., 2018). Moreover, the burst-release effect is also related to the concentration of NPs used for drug release study. At low NPs concentration, the water molecules in PBS solution could penetrate the NPs more quickly compared to high NPs concentration, causing rapidly degradation of the NPs (Kızılbey, 2019). The controlled-release behavior of polymeric NPs due to the hydrophobic interaction between the polymer matrix and OMF drug offered a benefit in the slow sustained-release of the therapeutic agent over a studied period (Wischke and Schwendeman, 2008).

Moreover, the morphological analysis of OMF@NPs-anti-FAP was performed by field emission scanning electron microscopy (FESEM) and transmission electron microscopy (TEM). The FESEM and TEM images in Fig. 1E unveiled the homogeneity in both size and shape of the synthesized nanoparticles, showing spherical NPs with an actual size of 241.18 ± 36.01 nm.

### 3.2 Cytotoxicity test

**Fig. 2:**
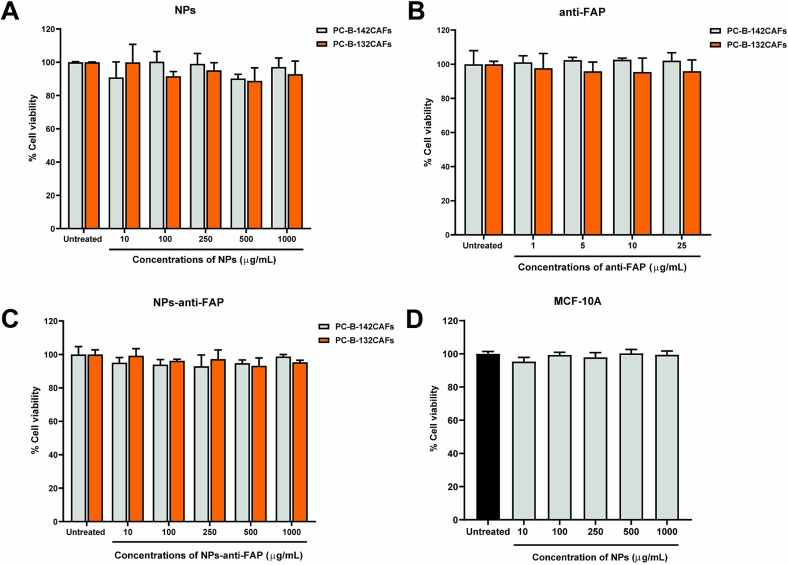
Cytocompatibility study of NPs, the anti-FAP monoclonal antibody, NPs-anti-FAPs on different cell types. (A-C) Cell viability of PC-B-142CAFs and PC-B-132CAFs after treatment with different concentrations of NPs, the anti-FAP monoclonal antibody, and NPs-anti-FAP at 24 h. (D) Cell viability of MCF-10A after treatment with different concentrations of NPs for 24 h.

In terms of safety, the biocompatibility of the nanoparticles to the cells was first verified. According to the results in Fig. 2A, the NPs were shown to be non-toxic to the cancer-associated fibroblasts (CAFs) with both low FAP-expressed PC-B-142CAFs and high FAP expressed PC-B-132CAFs at all concentrations. Moreover, both solution (anti-FAP) and particulate form of the monoclonal antibody (NPs-anti-FAP) also testified to their safety. The cell viability of CAFs shown in Fig. 2B and Fig. 2C proved that the anti-FAP monoclonal antibody did not cause any toxicity to the cells, albeit in the particulate formulation. This result confirmed that anti-FAP antibody would only function as a targeting moiety. The therapeutic effect would be attributed to the OMF drug only. Additionally, MCF-10A, representative of normal breast cells, was also tested with the NPs. Not surprisingly, the NPs did not show any toxicity to the cells as shown in Fig. 2D, confirming the biocompatibility of the carrier.

### 3.3 Cellular internalization

**Fig. 3:**
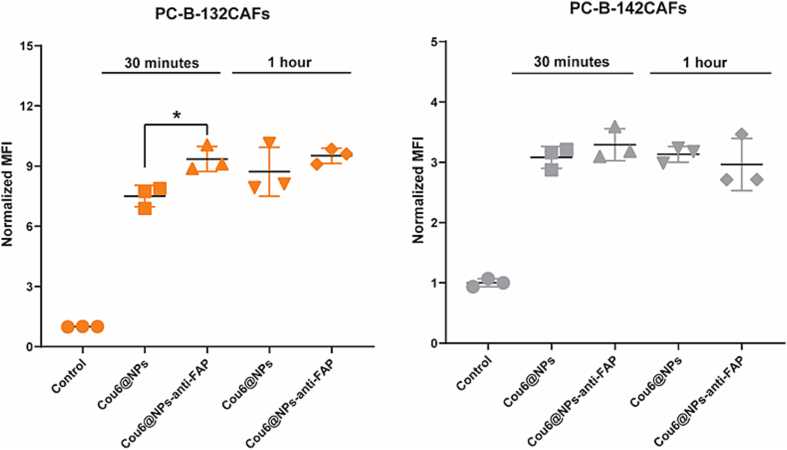
Cellular internalization analyzed by flow cytometry of Cou@NPs, and Cou6@NPs-anti-FAP at different times points in both high and low-FAP expressed CAFs (i.e. PC-B-132CAFs and PC-B-142CAFs, respectively). The data are presented as mean ± SD. A significant difference between samples is statistically identified with *: *p* < 0.05.

To elicit the function of an anti-FAP antibody as a CAFs-targeting ligand, anti-FAP-conjugated NPs loading with coumarin 6 (Cou6@NPs-anti-FAP), as a fluorescent NPs tracking probe, was treated with high- and low-FAP expressed CAFs, PC-B-132 and PC-B-142, respectively. The cellular internalization was conducted at 30 min and 1 h and analyzed by flow cytometry. Fig. 3 revealed the normalized mean fluorescence intensity (MFI) analyzed from flow cytometry. Regarding the MFI, the specific binding affinity between anti-FAP antibody and high-FAP expressed PC-B-132CAFs could be observed at 30 min of treatment while there was no difference between unconjugated- and antibody-conjugated NPs at longer time-points. This might be attributed to the cellular internalization through passive targeting effect (Kumari et al., 2023). Moreover, there was no significant degree of cellular internalization observed in low FAP-expressed cells among those particles, suggesting negligible effect of anti-FAP antibody on low-FAP expressed cells. This result implied that anti-FAP antibody specifically improved the targeting ability of NPs to the cells with high levels of FAP expression. It is consistent with a previous report confirming the specific targeting of anti-FAP antibody to the FAP-positive CAFs (Sitia et al., 2021).

### 3.4 Cytotoxicity study and Caspase-3/7 activity

**Fig. 4:**
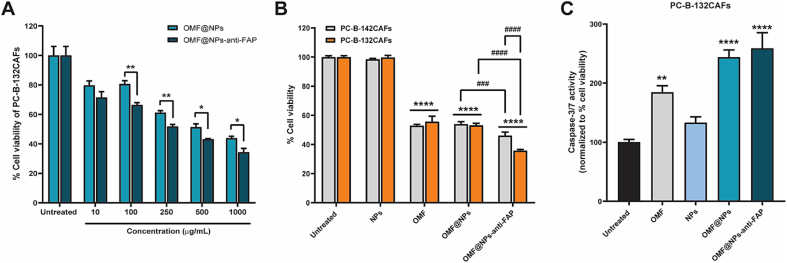
Effect of nanoparticles' concentration towards cell viability of PC-B-132CAFs. (A) Cytotoxicity of OMF@NPs and OMF@NPs-anti-FAP with different concentrations on the viability of PC-B-132CAFs. Inhibitory effect of free OMF, OMF-loaded NPs with and without anti-FAP antibody conjugation on PC-B-142CAFs and PC-B-132CAFs. (B) Cell viability of PC-B-142CAFs and PC-B-132CAFs and (C) Caspase-3/7 activity of PC-B-132CAFs after treatment with different formulations of nanoparticles at a concentration of 1 mg/mL (equivalent to 50 μM of OMF) for 24 h. The presented data are mean ± SD (*n* = 3). The significant difference between sample and untreated cell control is statistically identified with *:*p* < 0.05, **:*p* < 0.01, ****:*p* < 0.0001. The significant difference between the two samples is statistically identified with ###:*p* < 0.001, ####:*p* < 0.0001.

Next, we evaluated the cytotoxic effect of OMF drugs against CAFs. Firstly, the cytotoxicity of OMF-loaded NPs both with and without anti-FAP antibody at different concentrations from 10 μg/mL to 1000 μg/mL was determined against PC-B-132CAFs. As shown in Fig. 4A, the cytotoxicity of the nanoformulations was found to be concentration-dependent. Notably, the presence of the anti-FAP antibody resulted in a lower number of viable cells, indicating enhanced accumulation and targeted drug delivery compared to the unconjugated formulations. Then, the cytotoxicity of the fabricated nanosystems including free OMF, OMF@NPs, and OMF@NPs-anti-FAP to both CAFs was determined through cell viability. The equivalent amount of OMF in both free form and encapsulated ones was tested with the CAFs for 24 h. Fig. 4B demonstrated that all formulations caused significant cell death in both PC-B-142CAFs and PC-B-132CAFs by decreasing more than 40 % of viable cells, regardless of the level of FAP expression. This cytotoxicity was ascribed to the existence of the OMF compound. Interestingly, the OMF@NPs could inhibit the cells' proliferation to the same extent as that of the free form of OMF, although the NPs only released half of the loaded amount of OMF regarding the result in Fig. 1D. Thus, this result emphasizes the utilization of NPs as a drug carrier in minimizing toxicity caused by overdose. In addition, nanoparticles with the presence of the targeting moiety, OMF@NPs-anti-FAP, could visibly enhance the mortality of high FAP expressed PC-B-132CAFs, compared to the low FAP expressed one. This enhanced killing effect was shown to be due to the benefit of targeting ligands.

As it could be seen, OMF could be the key to causing cell death. We then testified to caspase-3/7 activity of high FAP-expressed CAFs after being treated with different platforms for 24 h. Caspase-3/7 activity of PC-B-132CAFs cells could be observed among samples possessing OMF compound (Fig. 4C). OMF-delivered nanoparticles, OMF@NPs, and OMF@NPs-anti-FAP, exhibited caspase-3/7 activity of 1.3- and 1.4-fold compared to free OMF, respectively. This caspase-3/7 activity was related to the stage of the apoptotic pathway (Shim et al., 2017). Conclusively, OMF evidently caused apoptotic cells.

### 3.5 Determination of FAP expression on cells' surface

**Fig. 5:**
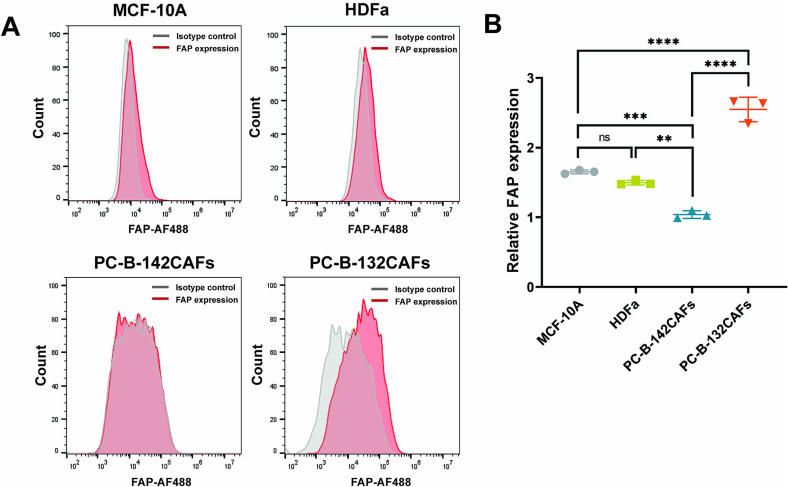
FAP expression analysis of different cell types determined by flow cytometry. (A) Histogram analyzed from flow cytometry showing the degree of FAP expression on MCF-10A, HDFa, PC-B-142CAFs and PC-B-132CAFs. (B) Relative mean fluorescence intensity of FAP expression on MCF-10A, HDFa, PC-B-142CAFs and PC-B-132CAFs. The presented data are mean ± SD (n = 3). Significant difference in two samples is statistically identified with **: *p* < 0.01, ***: *p* < 0.001, ****: *p* < 0.0001.

FAP is our prime target in this work. Thus, we investigated the level of FAP expression on different cells, including MCF-10A, HDFa, PC-B-142CAFs, and PC-B-132CAFs by using flow cytometry. Fig. 5A displayed the histograms analyzed from flow cytometry showing the degree of FAP expression on different cell types. Additionally, the relative mean fluorescence intensity obtained from flow cytometry revealed in Fig. 5B. Regarding those results, it could be seen that PC-B-132CAFs possessed a high level of FAP, while the others displayed a relatively low extent of FAP.

### 3.6 Cytotoxicity of drug-loaded NPs in 3D spheroid models

**Fig. 6:**
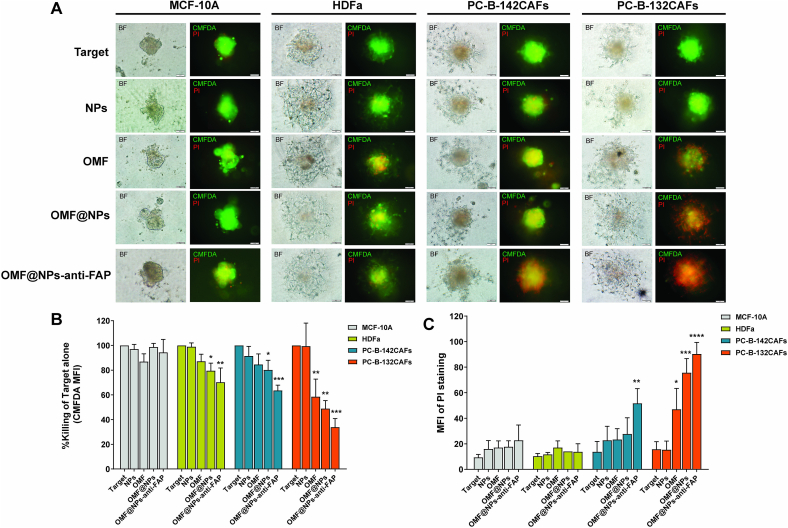
Cytoxicity study of different formulations of NPs in 3D spheroid model of MCF-10A, HDFa, PC-B-142CAFs and PC-B-132CAFs. (A) Cytotoxicity test of different therapeutic platforms in 3D spheroid observed under an inverted fluorescence microscope. Green; CMFDA staining represents live cells. Red; PI staining represents dead cells. (B) Quantitative evaluation of cytotoxicity shown as % killing of different targets after being treated with different formulations of NPs. (C) Mean fluorescence intensity of PI staining. The presented data are mean ± SD (*n* = 3). The significant difference between sample and target cell control is statistically identified with *: *p* < 0.05, **: *p* < 0.01, ***: *p* < 0.001, ****: *p* < 0.0001. (For interpretation of the references to colour in this figure legend, the reader is referred to the web version of this article.)

To verify the specificity and penetrating ability of CAFs-targeting nanoparticles, herein, we constructed 3D-spheroid models of low FAP-expressed cells, such as MCF-10A, HDFa, and PC-B-142CAFs and high FAP-expressed cells of PC-B-132CAFs. Different therapeutic platforms of free OMF, NPs, OMF@NPs, and OMF@NPs-anti-FAP (at an equivalent amount of OMF) were co-cultured with those 3D-spheroid cells. Subsequently, the percentage of killing was evaluated after 24-h of incubation. Fig. 6A displayed the images of live and dead cells, which were stained with 5-chloromethylfluorescein diacetate (CMFDA) and propidium iodide (PI), respectively. The fluorescence intensity of CMFDA was convertly quantified as % cytotoxicity and the mean fluorescence intensity of PI was also related to the degree of dead cells as displayed in Fig. 6B and Fig. 6C, respectively. Regarding the results, it was clearly observed that the cells with low expression of FAP (i.e. MCF-10A, HDFa, and PC-B-142CAFs) showed slight toxicity. In contrast to those cells, PC-B-132CAFs displayed severe toxicity after being treated with nanoparticles. In particular, the OMF@NPs-anti-FAP had a more susceptible killing effect on the PC-B-132CAFs than the nanoparticles without targeting ligands. This result is correlative to the amount of FAP expression found in Fig. 5. In addition, due to the aid of CAFs-targeting anti-FAP, the nanoparticles not only demonstrated high toxicity against targeted CAF,s but also performed an enhanced penetration into the core of PC-B-132CAFs compared to the unmasked one, OMF@NPs. This enhanced killing effect was certainly attributed to anti-FAP antibody, assuring the role of anti-FAP antibody as CAFs targeting ligand. Nevertheless, OMF in free form was not as effective as in particulate form. This might be due to the poor permeability of hydrophobic drugs. Therefore, our nanosystems could serve as a potent therapeutic platform against FAP-positive CAFs.

## 4 Conclusion

In this work, we introduced a novel platform of OMF-loaded NPs for an enhanced CAF targeting. The results confirmed that the OMF was a potent drug for killing CAFs through apoptotic pathways. In addition, a particulate form of OMF, OMF@NPs, could be beneficial over free formulation in the aspect of enhanced cell penetration. Nevertheless, the killing effect of drug-loaded NPs could be significantly improved, thereby conjugating CAFs-targeting ligand. The killing effect and penetrating capacity of OMF@NPs-anti-FAP were clearly verified in the 3D-spheroid model. The FAP-positive CAFs were susceptible to the nanosystems, showing a high level of dead cells, compared to the other cells. Therefore, our synthesized CAFs-targeting nanoplatforms could hold a potent candidate for an effective approach for breast cancer treatment.

## CRediT authorship contribution statement

**Kamonlatth Rodponthukwaji:** Writing – original draft, Methodology, Investigation, Formal analysis. **Suyanee Thongchot:** Methodology, Investigation, Formal analysis. **Suttikiat Deureh:** Methodology, Conceptualization. **Tanva Thongkleang:** Methodology, Investigation. **Mattika Thaweesuvannasak:** Methodology. **Kornrawee Srichan:** Methodology. **Chatchawan Srisawat:** Validation. **Peti Thuwajit:** Supervision, Conceptualization. **Kytai T. Nguyen:** Writing – review & editing. **Kwanruthai Tadpetch:** Writing – review & editing, Funding acquisition. **Chanitra Thuwajit:** Writing – review & editing, Supervision, Funding acquisition, Conceptualization. **Primana Punnakitikashem:** Writing – review & editing, Validation, Supervision, Funding acquisition, Conceptualization.

## Declaration of competing interest

All the authors read the manuscript before submission and declared that they have no conflict of interest.

## Data Availability

The data that support the findings of this study are available from the corresponding author, upon reasonable request.

## References

[bb0005] Breastcancer.org (2023). Breast Cancer Facts and Statistics. https://www.breastcancer.org/facts-statistics.

[bb0010] Calzoni E., Cesaretti A., Polchi A., Di Michele A., Tancini B., Emiliani C. (2019). Biocompatible Polymer Nanoparticles for Drug delivery applications in Cancer and Neurodegenerative Disorder Therapies. J Funct Biomater.

[bb0015] Chuangchot N., Jamjuntra P., Yangngam S., Luangwattananun P., Thongchot S., Junking M., Thuwajit P., Yenchitsomanus P.-T., Thuwajit C. (2023). Enhancement of PD-L1-attenuated CAR-T cell function through breast cancer-associated fibroblasts-derived IL-6 signaling via STAT3/AKT pathways. Breast Cancer Res..

[bb0020] Dana P., Thumrongsiri N., Tanyapanyachon P., Chonniyom W., Punnakitikashem P., Saengkrit N. (2023). Resveratrol Loaded Liposomes Disrupt Cancer Associated Fibroblast Communications within the Tumor Microenvironment to Inhibit Colorectal Cancer Aggressiveness. Nanomaterials.

[bb0025] Dang Thi T.A., Vu Thi T.H., Thi Phuong H., Ha Nguyen T., Pham The C., Vu Duc C., Depetter Y., Van Nguyen T., D'Hooghe M. (2015). Synthesis and anticancer properties of new (dihydro)pyranonaphthoquinones and their epoxy analogs. Bioorg. Med. Chem. Lett..

[bb0030] Elmowafy E.M., Tiboni M., Soliman M.E. (2019). Biocompatibility, biodegradation and biomedical applications of poly(lactic acid)/poly(lactic-co-glycolic acid) micro and nanoparticles. J. Pharm. Investig..

[bb0035] Gandhi S., Shende P. (2024). Anti-CD64 Antibody-Conjugated PLGA Nanoparticles Containing Methotrexate and Gold for Theranostics Application in Rheumatoid Arthritis. AAPS PharmSciTech.

[bb0040] Glentis A., Oertle P., Mariani P., Chikina A., El Marjou F., Attieh Y., Zaccarini F., Lae M., Loew D., Dingli F., Sirven P., Schoumacher M., Gurchenkov B.G., Plodinec M., Vignjevic D.M. (2017). Cancer-associated fibroblasts induce metalloprotease-independent cancer cell invasion of the basement membrane. Nat. Commun..

[bb0045] Guo Z., Zhang H., Fu Y., Kuang J., Zhao B., Zhang L., Lin J., Lin S., Wu D., Xie G. (2023). Cancer-associated fibroblasts induce growth and radioresistance of breast cancer cells through paracrine IL-6. Cell Death Dis..

[bb0050] Gutiérrez-Valenzuela C.A., Esquivel R., Guerrero-Germán P., Zavala-Rivera P., Rodríguez-Figueroa J.C., Guzmán-Z R., Lucero-Acuña A. (2018). Evaluation of a combined emulsion process to encapsulate methylene blue into PLGA nanoparticles. RSC Adv..

[bb0055] Hazeri Y., Samie A., Ramezani M., Alibolandi M., Yaghoobi E., Dehghani S., Zolfaghari R., Khatami F., Zavvar T., Nameghi M.A., Abnous K., Taghdisi S.M. (2022). Dual-targeted delivery of doxorubicin by mesoporous silica nanoparticle coated with AS1411 aptamer and RGDK-R peptide to breast cancer in vitro and in vivo. J Drug Deliv Sci Technol.

[bb0060] Kızılbey K. (2019). Optimization of Rutin-Loaded PLGA Nanoparticles Synthesized by Single-Emulsion Solvent Evaporation Method. ACS Omega.

[bb0065] Kumari M., Acharya A., Krishnamurthy P.T. (2023). Antibody-conjugated nanoparticles for target-specific drug delivery of chemotherapeutics. Beilstein J. Nanotechnol..

[bb0070] Lee N.K., Wang C.-P.J., Lim J., Park W., Kwon H.-K., Kim S.-N., Kim T.-H., Park C.G. (2021). Impact of the conjugation of antibodies to the surfaces of polymer nanoparticles on the immune cell targeting abilities. Nano Converg.

[bb0075] Lemos A.S.O., Campos L.M., Melo L., Guedes M., Oliveira L.G., Silva T.P., Melo R.C.N., Rocha V.N., Aguiar J.A.K., Apolônio A.C.M., Scio E., Fabri R.L. (2018). Antibacterial and Antibiofilm Activities of Psychorubrin, a Pyranonaphthoquinone Isolated from Mitracarpus frigidus (Rubiaceae). Front. Microbiol..

[bb0080] Li J.J., Tsang J.Y., Tse G.M. (2021). Tumor Microenvironment in Breast Cancer-Updates on Therapeutic Implications and Pathologic Assessment. Cancers (Basel).

[bb0085] Lou S., Ye J.Y., Li K.Q., Wu A. (2012). A gold nanoparticle-based immunochromatographic assay: the influence of nanoparticulate size. Analyst.

[bb0090] Mady O.Y., Thabit S.M., Abo Elnasr S.E., Hedaya A.A. (2023). An industrial procedure for the intestinal permeability enhancement of acyclovir: in-vitro and histological evidence. Sci. Rep..

[bb0095] Misiak P., Niemirowicz-Laskowska K., Markiewicz K.H., Wielgat P., Kurowska I., Czarnomysy R., Misztalewska-Turkowicz I., Car H., Bielawski K., Wilczewska A.Z. (2023). Doxorubicin-loaded polymeric nanoparticles containing ketoester-based block and cholesterol moiety as specific vehicles to fight estrogen-dependent breast cancer. Cancer Nanotechnol..

[bb0100] Mohan A.K., M.M., Kumar TRS, Kumar GSV (2022). Multi-Layered PLGA-PEI Nanoparticles Functionalized with TKD Peptide for Targeted delivery of Pep5 to Breast Tumor Cells and Spheroids. Int. J. Nanomedicine.

[bb0105] Monteran L., Erez N. (2019). The Dark Side of Fibroblasts: Cancer-Associated Fibroblasts as Mediators of Immunosuppression in the Tumor Microenvironment. Front. Immunol..

[bb0110] Punuch K., Wongwan C., Jantana S., Somboonyosdech C., Rodponthukwaji K., Kunwong N., Nguyen K., Sirivatanauksorn V., Srisawat C., Punnakitikashem P. (2022). Study of siRNA delivery via Polymeric Nanoparticles in Combination with Angiogenesis Inhibitor for The Treatment of AFP-Related Liver Cancer. Int. J. Mol. Sci..

[bb0115] Rao M.S., Gupta R., Liguori M.J., Hu M., Huang X., Mantena S.R., Mittelstadt S.W., Blomme E.A.G., Van Vleet T.R. (2019). Novel Computational Approach to Predict Off-Target Interactions for Small Molecules. Front Big Data.

[bb0120] Rodponthukwaji K., Pingrajai P., Jantana S., Taya S., Duangchan K., Nguyen K.T., Srisawat C., Punnakitikashem P. (2024). Epigallocatechin Gallate Potentiates the Anticancer effect of AFP-siRNA-Loaded Polymeric Nanoparticles on Hepatocellular Carcinoma Cells. Nanomaterials.

[bb0125] Saha T., Fojtů M., Nagar A.V., Thurakkal L., Srinivasan B.B., Mukherjee M., Sibiyon A., Aggarwal H., Samuel A., Dash C., Jang H.L., Sengupta S. (2024). Antibody nanoparticle conjugate–based targeted immunotherapy for non–small cell lung cancer. Sci. Adv..

[bb0130] Saisin S., Panthong K., Hongthong S., Kuhakarn C., Thanasansurapong S., Chairoungdua A., Suksen K., Akkarawongsapat R., Napaswad C., Prabpai S., Nuntasaen N., Reutrakul V. (2023). Pyranonaphthoquinones and Naphthoquinones from the Stem Bark of Ventilago harmandiana and their Anti-HIV-1 activity. J. Nat. Prod..

[bb0135] Service, N.H (2022). Treatment: Breast cancer in women. https://www.nhs.uk/conditions/breast-cancer/treatment/.

[bb0140] Shim M.K., Yoon H.Y., Lee S., Jo M.K., Park J., Kim J.-H., Jeong S.Y., Kwon I.C., Kim K. (2017). Caspase-3/−7-specific Metabolic Precursor for Bioorthogonal Tracking of Tumor Apoptosis. Sci. Rep..

[bb0145] Shipunova V.O., Sogomonyan A.S., Zelepukin I.V., Nikitin M.P., Deyev S.M. (2021). PLGA Nanoparticles decorated with Anti-HER2 Affibody for Targeted delivery and Photoinduced Cell Death. Molecules.

[bb0150] Sitia L., Bonizzi A., Mazzucchelli S., Negri S., Sottani C., Grignani E., Rizzuto M.A., Prosperi D., Sorrentino L., Morasso C., Allevi R., Sevieri M., Silva F., Truffi M., Corsi F. (2021). Selective Targeting of Cancer-Associated Fibroblasts by Engineered H-Ferritin Nanocages Loaded with Navitoclax. Cells.

[bb0155] Sun S.B., Liu P., Shao F.M., Miao Q.L. (2015). Formulation and evaluation of PLGA nanoparticles loaded capecitabine for prostate cancer. Int. J. Clin. Exp. Med..

[bb0160] Tacar O., Sriamornsak P., Dass C.R. (2013). Doxorubicin: an update on anticancer molecular action, toxicity and novel drug delivery systems. J. Pharm. Pharmacol..

[bb0165] Tang T., Gong Y., Gao Y., Pang X., Liu S., Xia Y., Liu D., Zhu L., Fan Q., Sun X. (2023). A pH-responsive liposomal nanoplatform for co-delivery of a Pt(IV) prodrug and cinnamaldehyde for effective tumor therapy. Front. Bioeng. Biotechnol..

[bb0170] Vijitphan P., Rukachaisirikul V., Muanprasat C., Iawsipo P., Panprasert J., Tadpetch K. (2019). Unified synthesis and cytotoxic activity of 8-O-methylfusarubin and its analogues. Org. Biomol. Chem..

[bb0175] Wischke C., Schwendeman S.P. (2008). Principles of encapsulating hydrophobic drugs in PLA/PLGA microparticles. Int. J. Pharm..

[bb0180] Xin L., Gao J., Zheng Z., Chen Y., Lv S., Zhao Z., Yu C., Yang X., Zhang R. (2021). Fibroblast Activation Protein-α as a Target in the Bench-to-Bedside Diagnosis and Treatment of Tumors: a Narrative Review. Front. Oncol..

[bb0185] Yoshida G.J. (2020). Regulation of heterogeneous cancer-associated fibroblasts: the molecular pathology of activated signaling pathways. J. Exp. Clin. Cancer Res..

[bb0190] Zhang Z., Wang X., Li B., Hou Y., Cai Z., Yang J., Li Y. (2018). Paclitaxel-loaded PLGA microspheres with a novel morphology to facilitate drug delivery and antitumor efficiency. RSC Adv..

